# Supplementary material for: Venomix: a simple bioinformatic pipeline for identifying and characterizing toxin gene candidates from transcriptomic data
Source: PeerJ. 2018 Jul 31;6:e5361. doi: 10.7717/peerj.5361 (PMC6074769; doi:10.7717/peerj.5361)
Supplement: Supplemental Information 3 [file peerj-06-5361-s003.gz › FinalOutput_E-6/Toxins/Snake_venom_metalloproteinase_BnP1_2/finaltree.pdf]

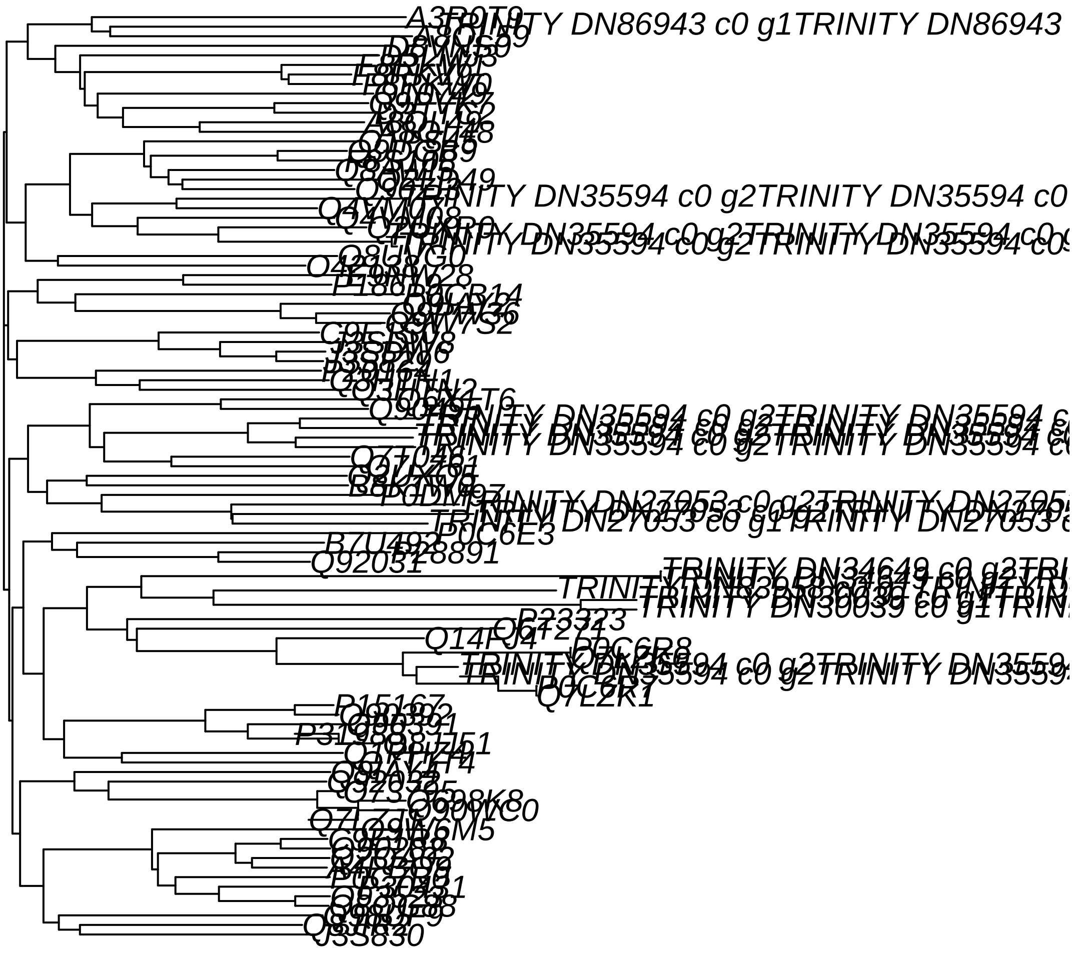
A3P0T9
TRINITY DN86943 c0 g1TRINITY DN86943
TRINITY DN35594 c0 g2TRINITY DN35594 c0
P0C6E3
B7U492
P28891
Q92031
P23323
Q14FJ4
P0C6R8
Q7L2R1
P15167
Q7LZI6
J3S830
